# The course of complaints of arm, neck and/or shoulder: a cohort study in a university population participating in work or study

**DOI:** 10.1186/s12891-018-2116-5

**Published:** 2018-06-30

**Authors:** Vivian E. J. Bruls, Nicole W. H. Jansen, Sander M. J. van Kuijk, IJmert Kant, Caroline H. G. Bastiaenen

**Affiliations:** 1 Department of Epidemiology, CAPHRI Care and Public Health Research Institute, Maastricht University, PO Box 616, 6200 MD Maastricht, The Netherlands; 2 Department of Clinical Epidemiology and Medical Technology Assessment (KEMTA), Maastricht University Medical Centre+, Maastricht, The Netherlands

**Keywords:** Complaints of arm, neck or shoulder, CANS, Course, Screened population, Prevention, Selection effects, University population, Students, Employees

## Abstract

**Background:**

Not much is known about the characteristics, course and prognosis of complaints of arm, neck and/or shoulder that have not been caused by a trauma or systemic disease (CANS), in a screened population. This study aims to: (1) describe personal and complaint characteristics in a screened population; (2) describe the course during one-year follow-up, in terms of the three different domains of functioning of the International Classification of Functioning, Disability and Health (ICF); and (3) to explore prognostic factors for the different domains of functioning at one-year follow-up. Additionally, this study aims to investigate the manifestation of selection effects (i.e. tertiary selection effects), in order to understand their impact on the interpretation of results.

**Methods:**

A cross-sectional survey was conducted in a university population. Survey respondents who fulfilled eligibility criteria were asked to participate in a longitudinal cohort study. The course of CANS was assessed in terms of the three ICF domains of functioning. Possible prognostic factors across the different components of the ICF were selected to investigate their influence on outcome at one-year follow-up. Non-response analyses were performed to investigate the presence of tertiary selection effects.

**Results:**

The results revealed a population with relatively mild complaints at baseline, and a relatively stable course during follow-up. Because of the small change in scores between baseline and follow-up measurements, examination of prognostic factors was not feasible. The results of the non-response analyses revealed some indications for the potential presence of tertiary selection effects, which may imply that the results obtained are a slight overestimation of the true results.

**Conclusion:**

The results of this study demonstrate mild complaints at baseline and an overall stable course during one-year follow-up. Since selection effects cannot be ruled out, the true course might possibly be somewhat less favourable than our results suggest.

## Background

Upper extremity disorders (UEDs) are a worldwide health problem resulting in a negative impact on a person’s wellbeing, as well as high costs to society [1]. A survey in the general Dutch population on the prevalence of complaints of arm, neck and/or shoulder, not caused by a trauma or systemic disease (CANS), revealed a 12-month prevalence of 37%, and a point prevalence of 26% [2]. Moreover, it has been shown that a considerable number of CANS become chronic [3].

The potential impact of these complaints on both private and working life emphasises the need for an effective intervention. However, the results of a recent Cochrane review on conservative interventions for treating chronic work-related CANS revealed no consistent evidence for any specific treatment on pain, recovery, disability or sick leave [4]. With ongoing clinical uncertainty regarding an effective treatment strategy for CANS, it is hypothesised that an intervention aimed at beginning and mild complaints, before disability or sick leave occurs, would be more effective in preventing chronic problems. When considering an early preventive intervention for CANS aimed at individuals with beginning and relatively mild complaints, insight in the course and prognosis of CANS is essential.

Until now, studies on the course and prognosis of CANS have been largely undertaken in healthcare settings and not much is known regarding the natural course of the disease [5, 6]. It should be noted that patients with CANS who contact healthcare professionals display several characteristics inherent to this particular population. Firstly, healthcare users are known to report more severe complaints, worse general health, more limitations in daily living, and more sickness absence, compared to non-healthcare users [2]. Second, a study population which is recruited in healthcare settings displays help-seeking behaviour. According to literature, the decision to seek help from healthcare professionals is associated with various socio-demographic and attitudinal factors [7, 8]. Therefore, the findings of these studies cannot be readily generalised to a population with relatively mild complaints who have not necessarily contacted a healthcare professional so far. Consequently, in a screened population the course and prognostic factors may be different.

A study by Picavet et al. on musculoskeletal complaints in the general Dutch population [3] found that approximately 70% of those with complaints indicated that they experience mild complaints, and between 33 and 42% contacted a healthcare professional. Hence, these numbers suggest there is a large group of individuals with CANS beyond the confines of healthcare settings, for which information on the course and prognosis is lacking. Insight in the course and prognosis of the health state in such a population when screened will provide valuable information of a far wider group within the total population of people with CANS.

**Fig. 1:**
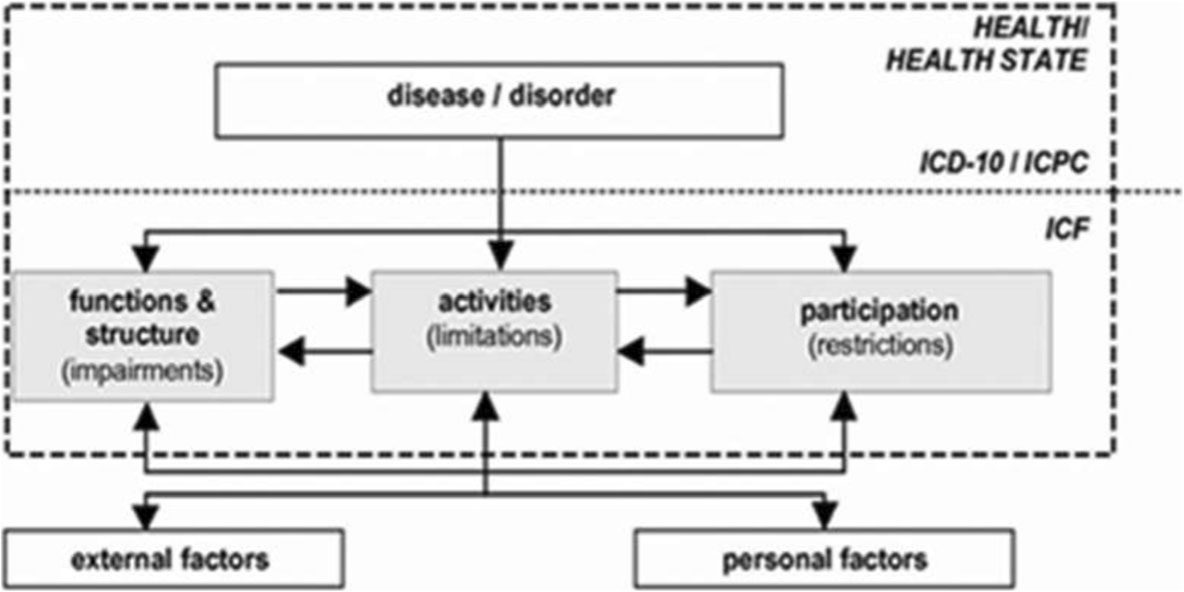
Scheme of the ICF (WHO, 2001). Grey areas represent the different domains of functioning

**Fig. 2:**
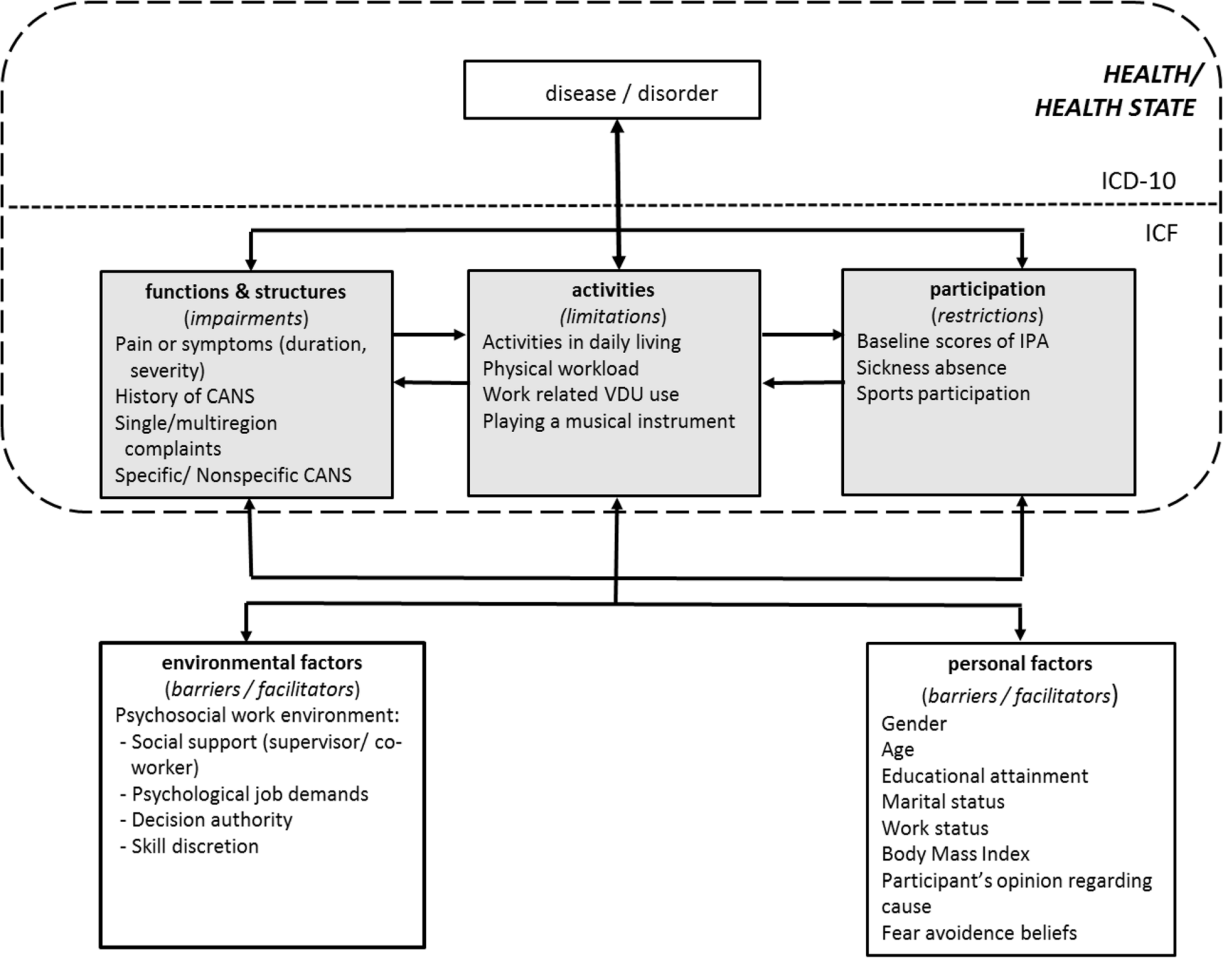
ICF-based conceptual model applied to CANS, comprising illustrations of the different domains of functioning in the context of personal and environmental factors

To describe an individual’s health state over the course of time, one should keep in mind that a person’s health state comprises the absence or presence of a disease or disorder but also includes the person’s functioning. There is a complex interaction between health state (including functioning) and contextual factors (i.e. environmental and personal factors) [9]. These factors interact with an individual with a certain health state and determine the level and extent of the individual’s functioning. In other words, the level of experienced symptoms or the extent of limitations in functioning as an entity do not necessarily equate to the health state of an individual. For example, person A experiences impairments in body functions, but does not perceive any restrictions in participation due to adaptations in (work) environment. On the other hand, person B does experience severe restrictions in participation, due to stigmatisation because of having a certain disease, although no evident impairments are present. A framework which fits the comprehensive concept of health state by categorising it in disease/disorder and different components of functioning in the context of personal and environmental factors is the International Classification of Functioning, Disability and Health (ICF) (Fig. 1) [9]. The health- and health-related states can be described by using this classification [9]. Note that the ICF is not a diagnosis-based system, but can be used as a conceptual framework for functioning even if a diagnosis is absent. Particularly in the case of CANS, which includes both diagnosable and non-diagnosable conditions according to the definition of the CANS model [10], a specific diagnosis is frequently lacking. Consequently, the ICF may offer an excellent tool to describe the course of CANS and to guide and map possible prognostic factors which influence the individuals’ functioning at follow-up. When designing the current study, an ICF-based conceptual model for studying course and prognostic factors in CANS was developed by selecting appropriate concepts for the different domains of functioning, applied to CANS, and identifying putative prognostic factors from the literature (Fig. 2). To date, literature on the prognosis of CANS reveals a broad range of possible prognostic factors. However, the majority of these prognostic factors could not be confirmed by a recent systematic review [5]. In addition, since most studies on the prognosis of CANS have taken place in healthcare populations as mentioned above, it remains unclear which factors influence the course of CANS in individuals of a general population.

The aims of the present study are to obtain insights in the characteristics, course and prognosis of CANS in a screened population by: (1) describing personal and complaint characteristics of a screened population with self-reported CANS, divided into subgroups for < 3 months and ≥ 3 months duration of complaints; (2) describing the course of CANS during one-year follow-up in terms of the three different domains of functioning according to the ICF (impairments in body structures, limitations in activities, and restrictions in participation); and (3) exploring the influence of possible prognostic factors on the three domains of functioning, at one-year follow-up.

When investigating the characteristics, course and prognostic factors, the presence of selection effects should be taken into account, as it is well known that selection effects are likely to occur in longitudinal studies. Different manifestations of selection effects are possible. For example, a primary selection effect which arises when individuals who already experience CANS decide not to engage in physically or psychologically demanding work, and will therefore not be included in a longitudinal study of a working population. A secondary selection effect which takes place when individuals with CANS adapt their work tasks over time in order to be less exposed to adverse work demands, or leave the workplace which is responsible for inducing their physical complaints, prematurely For example, if they experience that their current tasks conflict with their physical complaints, they switch to work with lower exposure levels. Finally, a tertiary selection effect may take place when, due to selective non-response during the follow-up, only a selected group is included in the study analyses, resulting in a bias of the true results. For example, those with more severe complaints do not respond due to functional limitations.

To determine whether the results of this study are a possible over- or underestimation of the true results in the study population, this study also aims to consider the mechanism of selective non-response during follow-up when interpreting the results, in order to understand the impact of potential selection effects.

## Methods

### Design, setting and participants

**Fig. 3:**
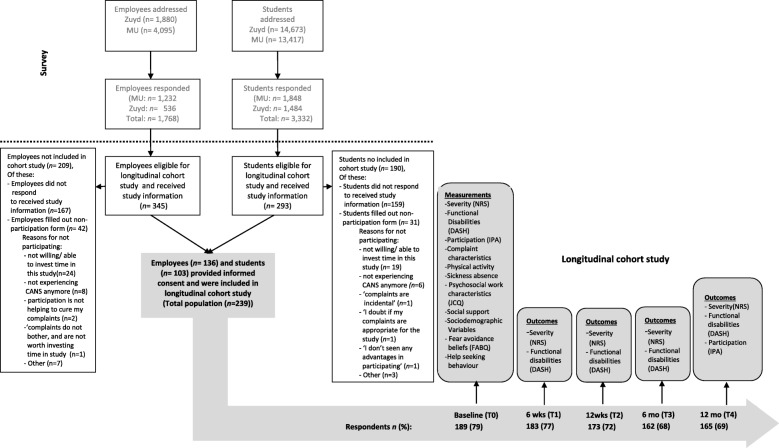
Design of the CANS Cohort Study, depicting the participant flow diagram and follow-up measurements. Zuyd = Zuyd University of Applied Sciences; MU = Maastricht University; NRS=Numeric Rating Scale; IPA = Impact on Participation and Autonomy; JCQ = Job Content Questionnaire; FABQ = Fear Avoidance Beliefs Questionnaire; DASH = Disabilities of Arm, Shoulder, Hand questionnaire; T = Time point; wks = weeks; mo = months

This longitudinal cohort study was embedded in the *CANS Cohort Study* [11]. Prior to conducting a longitudinal cohort study, a large cross-sectional survey aimed at screening for CANS was conducted among students and employees at two large universities in the southern region of the Netherlands, Maastricht University and Zuyd University of Applied Sciences. In total, 5975 employees and 28,090 registered students were invited to participate in this survey. The primary aim of the survey questionnaire was to gain insight in the prevalence of CANS within the university population. Figure 3 depicts the participant flow diagram.

Participants for the longitudinal cohort study were recruited from the survey respondents who indicated experiencing CANS (*n* = 1396). Participant recruitment took place from September 2013 through December 2013. Inclusion criteria were: respondents who indicated experiencing CANS at that particular moment or in the preceding 3 months. Respondents were excluded when complaints were caused by trauma (e.g. fracture, dislocation), malignancy, amputation, prosthesis, congenital defect or a co-morbidity causing severe disability in daily life. Respondents who were pregnant were also excluded from participation (*n* = 3). Survey respondents who fulfilled the eligibility criteria were asked to participate in a follow-up study. Details of the study procedure are described elsewhere [11]. In total, *n* = 638 respondents fulfilled the eligibility criteria, of whom *n* = 239 provided informed consent. These 239 individuals entered the study as baseline population (T0) and were followed up for 1 year. Follow-up measurements were scheduled at 6 weeks (T1), 12 weeks (T2), 6 months (T3) and 12 months (T4) after baseline measurements. Timing of measurements and corresponding outcome measures are depicted in Fig. 3.

Ethical approval was granted by the Medical Ethics Committee of the University Hospital Maastricht and Maastricht University (METC 13–4-045), the Netherlands, and informed consent was obtained from all participants.

### Measurements

#### Descriptives of the study population

Personal characteristics gathered at baseline (T0) included: age, gender, paid work (yes/no), marital status, children in the household < 5 years old, body mass index (BMI) (calculated from self-reported weight and height), smoking behaviour (assessed by the question ‘Do you currently smoke tobacco?’ (no, never smoked; no, but I used to smoke now and then; no, but I used to smoke every day; yes, I smoke now and then; yes, I smoke every day), alcohol consumption (assessed by the question ‘Do you consume alcohol?’ (no, never or seldom; yes, now and then (< 3 drinks per week); yes, more than 3 drinks per week)), sports participation (assessed by asking participants whether they performed heavy physical activity which makes them sweat for at least two times per week for 30 min (yes/no)), meeting Dutch guidelines for physical activity [12] (30 min of moderate-intensity physical activity on at least 5 days per week (yes/no), use of Visual Display Unit (VDU) for > 2 h per day (yes/no). Physical activities during leisure time were assessed by six items which were modified from questions and scores applied in a study by Karels et al. [13]. These six items comprised housekeeping, taking care of chronic patients and/or disabled persons, do-it-yourself activities, gardening, visual display use, and handicrafts. Response options ranged from 0 (seldom/never) to 3 (always/often) for each item. Based on these items, two scores were calculated, namely ‘heavy physical load in leisure time’ (i.e. housekeeping, taking care of chronic patients and/or disabled persons, do-it-yourself activities, gardening) and ‘static physical load in leisure time’ (i.e. visual display use and handicrafts).

Psychosocial characteristics included social support, measured using the Dutch and English versions of the Social Support Scale (SOS) [14]. The scale contains 12 items scored from 1 (‘no, not at all’) to 5 (‘very clearly’). The total score ranges from 12 to 60. A higher score indicated more social support. Catastrophizing was measured using the Pain Catastrophizing Scale (PCS) [15, 16]. The PCS is a 13-item scale, with each item scored on a 5-point scale: 0 (not at all) to 4 (all the time). Fear avoidance beliefs were measured using the Fear Avoidance Beliefs Questionnaire (FABQ) [17]. This questionnaire consists of two subscales: the Physical Activity subscale (4 items, score range 0–24) and the Work subscale (6 items, score range 0–42). Higher scores on the FABQ are indicative of greater fear and avoidance beliefs.

Complaint characteristics gathered at baseline comprised: duration of current episode (< 1 month; 1- <  3 months; 3–6 months; > 6 months), history of CANS (no; yes, once before; yes, several times before), discomfort of complaints (no discomfort; regular discomfort; almost continuously).

Work variables for *employees* in the study population included: full-time work (working > 20 h per week)(yes/no), working less than 3 years in current job (yes/no), work-related complaints according to participant (yes/no), sickness absence due to CANS at this moment or in the past 3 months (no/yes: if yes: duration of sickness absence < 1 month or ≥ 1 month), physical workload was measured using the short version of the Physical Workload Questionnaire (PWQ) [18]. The items address force exertions, as well as static, dynamic and repetitive movements of the upper extremity. Each item is scored on a two-point scale (‘no’ scores 1 and ‘yes’ scores 2). Two separate scores were calculated, namely ‘heavy physical workload’ (7 items) and ‘static repetitive work’ (8 items). Higher scores indicate higher physical load. Psychosocial factors at work were measured using the Job Content Questionnaire (JCQ) [19]. The JCQ includes the following subscales: psychosocial job demands (5 items, score ranges from 5 to 20); decision authority (3 items, score ranges from 3 to 12) and skill discretion (6 items, score ranges from 6 to 24); supervisor support (4 items, score ranges from 4 to 16); and co-worker support (4 items, score ranges from 4 to 16). For each item, the response options were: 1 = strongly disagree, 2 = disagree, 3 = agree, 4 = strongly agree. For each scale, the overall score was calculated by summing the response scores of the individual items. Higher scores indicate higher job demands, decision authority, skill discretion, supervisor and co-worker support.

#### Course

To assess the course of CANS over one-year follow-up, appropriate measurement instruments were selected which were linked to the relevant ICF domain of functioning [9]:

1. *Functions and structures*
  Severity of complaints in the previous week was measured on an 11-point numerical rating scale (NRS-11) ranging from 0 to 10 using the following question: ‘*How would you describe the severity of your pain or complaints in the previous week on a scale from 0 (no pain/complaints) to 10 (intolerable pain/complaints)?*’. A score of 0 is considered as no pain or complaints, a score of 1–3 is considered as mild, a score of 4–6 as moderate, and a score of 7–10 is considered as severe pain or complaints [20]. The NRS-11 is a valid and reliable instrument for measuring pain intensity [21].
2. *Activities*
  Functional limitations of the arm, neck, shoulder or hand were measured using the Disability of Arm, Shoulder and Hand (DASH) questionnaire [22]. The question on sexual activities was excluded. This 29-item questionnaire included questions about symptoms as well as the ability to perform certain activities [22]. Each item was scored on a five-point Likert scale. Higher scores indicate more functional limitations. The DASH has been shown to be a reliable, valid and reproducible measurement tool for the shoulder, arm and hand region [23]. In addition, the DASH has shown acceptable validity and responsiveness for use in patients with non-traumatic neck complaints in addition to shoulder, arm, and hand complaints [24].
3. *Participation*
  Restrictions in participation and autonomy were measured using the Impact on Participation and Autonomy (IPA) questionnaire [25]. This 32-item questionnaire covers an individual’s perceived participation in five subscales. Each item has five response options, ranging from 0 (very good) to 4 (very poor) [25]. Higher scores represent poorer participation and autonomy. The five subscales are: autonomy indoors (7 items, total score range 0–28), family role (7 items, total score range 0–28), autonomy outdoors (5 items, total score range 0–20), social life and relationships (7 items, total range score 0–28), and work and education (6 items, total range score 0–24). The IPA questionnaire is valid, reliable and responsive to change [25–28].

#### Putative prognostic factors

Several putative prognostic variables were inventoried at baseline measurement (T0), to examine their influence on functioning at one-year follow-up. These variables were derived from the literature and classified according to the different domains of the ICF [9]. Because of the limited sample size (*n* = 239), the putative prognostic factors examined in this study were not exhaustive, but a selection of variables from the ICF-based conceptual model was operationalised for examination (Fig. 2).

##### Body functions and structures

Duration of the current episode at baseline; baseline scores on complaint severity (measured using NRS); single−/multi-region complaints (complaints were classified as multi-region if the participant indicated more than one region of symptoms, i.e. neck, shoulder and elbow); specific/non-specific CANS (participants were asked whether they were diagnosed with a specific complaint by a healthcare professional); history of CANS;

##### Activities

Baseline scores on functional limitations of the arm, neck, shoulder or hand (measured using the DASH) [22]; meeting the Dutch norm for healthy activity [12] (yes/no); physical activities during leisure time (assessed using six items as described above); physical workload (measured using the Physical Workload Questionnaire PWQ) [18].

##### Participation

Baseline scores on perceived restrictions in participation and autonomy were measured using the IPA [25]; sickness absence due to CANS at the moment of baseline measurements or in the preceding 3 months; sports participation (defined as performing heavy physical activity that makes you sweat for at least two times per week for a period of 30 min (yes/no)).

##### Environmental factors

Social support was measured using the Dutch and English versions of the Social Support Scale (SOS) [14]. Psychological work environment was operationalised using the Job Content Questionnaire (JCQ) [19]. The JCQ was only administered to those participants with a paid job.

##### Personal factors

Gender; age; educational attainment (defined as the highest educational level already completed: low = no education, primary school; medium = high school/General Educational Development test or college; High = Bachelor’s degree, Master’s degree, advanced graduate or PhD); marital status; work status (paid work yes/no); Body Mass Index (bodyweight in kilogrammes/(height in meters)^2^); participants’ opinion regarding cause (strain or overuse, unusual activities, sport injury, unknown); fear avoidance beliefs (measured using the Fear Avoidance Beliefs Questionnaire, FABQ) [17].

### Statistical analyses

Participant characteristics at baseline were summarised as means and standard deviations (SD), and median and range for normally and not normally distributed continuous variables, respectively, as well as absolute numbers and percentages for categorical variables. In addition to the description of participant characteristics for the total group of participants, a distinction was made between participants with duration of complaints at baseline lasting less than 3 months and participants with duration of complaints at baseline lasting 3 months or longer, in order to determine whether differences in participant characteristics were present between these two subgroups.

Descriptive statistics were used to describe the course of the complaints: mean reduction scores and standard deviations of the outcomes for complaints severity, functional limitations and impact on participation and autonomy were calculated.

Repeated measures analysis using linear mixed models was intended to qualify the relationships between the continuous dependent variables and predictive variables. Functioning in terms of complaint severity, functional limitations and impact on participation and autonomy at one-year follow-up, was considered as a dependent variable. Individual characteristics, complaint-specific characteristics, help-seeking behaviour, physical factors in work or leisure time, and psychological and social factors – all measured at baseline – were considered predictor variables. All available measurements of all participants were intended to be analysed without imputation for missing data, which is valid under the same assumptions as multiple imputation, and under less restrictive assumptions than simple imputation methods or complete case analysis [29].

All analysis was carried out using Statistical Package of Social Sciences, version 23, for Windows (SPSS Inc., Chicago, IL). *P*-values of < 0.05 were considered to indicate statistical significance.

## Results

### Descriptives of study population

Table 1 presents the characteristics of the study population at baseline. The mean age of the total study population was 35.5 years (SD = 13.3 years); 74% were women and 62% had a paid job. The majority of participants reported having experienced CANS previously (52%), and perceived regular (50%) or continuous (19%) discomfort from the complaints. At baseline, the mean score on the outcome ‘Severity of the complaints’ was 4, representing moderate severity [20]. The mean score on the primary outcome functional limitations (measured using DASH) was 15.05 (SD = 12.5), corresponding with mild to moderate functional limitations.

**Table 1:** Participant characteristics at baseline

	Total population (*n* = 239)	Subgroup Baseline duration < 3 mo (*n* = 133)^a^	Subgroup Baseline duration ≥3 mo (*n* = 101)^a^	*p-value*
Variables	Number of participants (%)^b^	%^b^	%^b^	
Participant characteristics				
Gender (female), n (%)	177 (74)	79.7	68.3	0.047
Age(years),mean (sd)	35.5 (13.3)	30.9 (12.17)	44.1 (11.2)	< 0.001
Body mass index (kg/m^2^), mean (sd)	23.9 (3.37)	23.4 (3.27)	24.5 (3.37)	0.026
Paid work, *n* (%)	149 (62)	39.8	91.1	< 0.001
Student status, *n* (%)	89 (38)	60.2	8.9	
Scientific staff, *n* (%)	60 (42.3)	49.1	38.2	0.205
Support staff, *n* (%)	82 (57.7)	50.9	61.9	
Marital status				< 0.001
Unmarried/ living alone, *n* (%)	68 (28)	49.5	19.7	
Married/ living together, *n* (%)	110 (46)	47.7	72.4	
Widow/ divorced, *n* (%)	9 (4)	2.8	7.9	
Unknown, *n* (%)	52 (22)			
Children in the household				0.312
No, *n* (%)	163 (68)	89.7	82.9	
Yes, one or more, *n* (%)	24 (10)	10.3	17.1	
Unknown, *n* (%)	52 (22)			
Health behaviour				
Currently smoking				0.063
No, I have never smoked, *n* (%)	119 (50)	72.0	53.9	
No, but I used to smoke now and then *n* (%),	22 (9.2)	11.2	13.2	
No, but I used to smoke every day, *n* (%)	24 (10)	10.3	17.1	
Yes, I smoke now and then, *n* (%)	3 (1)	1.9	1.3	
Yes, I smoke every day, *n* (%)	16 (7)	4.7	14.5	
Unknown, *n* (%)	55 (23)	–	–	
Alcohol consumption				0.134
No, never or seldom, *n* (%)	50 (21)	27.1	27.6	
Yes, now and then (< 3 drinks/week), *n* (%)	89 (37)	53.3	40.8	
Yes, > 3 drinks/ week, *n* (%)	45 (19)	19.6	31.6	
Unknown, *n* (%)	55 (23)	–	–	
Complaint characteristics				
Duration of current episode		–	–	–
< 1 month, *n* (%)	112 (46.9)			
1–3 months, *n* (%)	21 (8.8)			
3–6 months, *n* (%)	19 (7.9)			
> 6 months *n* (%)	82 (34.3)			
Unknown	5			
History of CANS				< 0.001
No, *n* (%)	22 (9.2)	19.4	9.3	
Yes, one time before, *n* (%)	17 (7.1)	20.9	3.1	
Yes, more times before, *n* (%)	125 (52.3)	59.7	87.6	
Discomfort of complaints				< 0.001
No discomfort, *n* (%)	1 (0.4)	1.4	0.0	
Regularly, *n* (%)	120 (50.2)	86.8	61.6	
Almost continuously, *n* (%)	46 (19.2)	11.8	38.4	
Baseline scores of outcome measures				
Complaints intensity in previous week (VAS) [0–10] mean (sd)	4.05 (2.28)	3.7	4.5	0.019
median [range]	4 [0–8]			
Disability (DASH) [0–100] mean (sd)	15.05 (12.5)	11.7 (10.5)	19.5 (13.8)	< 0.001
median [range]	13.36 [0.00–58.62]			
Impact on participation and autonomy (IPA),				
Autonomy indoors [0–4], mean (sd)	0.18 (0.33)	0.15 (0.30)	0.23 (0.37)	0.085
Family role [0–4] mean (sd),	0.51 (0.59)	0.38 (0.46)	0.66 (0.65)	0.001
Autonomy outdoors [0–4], mean (sd)	0.58 (0.62)	0.47 (0.53)	0.69 (0.69)	0.019
Social life and relationships [0–4], mean (sd)	0.45 (0.48)	0.41 (0.45)	0.50 (0.52)	0.214
Work and education [0–4], mean (sd)	0.86 (0.71)	0.71 (0.69)	1.03 (0.68)	0.005
Physical activity in leisure time				
Sports participation (yes),*n* (%)	117 (49)	48.9	42.0	0.297
NNGB (yes), *n* (%)	239 (100)	64.7	63.0	0.794
VDU use > 2 h/day (yes), *n* (%)	73 (31.5)	42.9	16.2	< 0.001
Heavy physical load (0–8), mean (sd)	3.05 (1.6)	2.47 (1.3)	2.92 (1.6)	0.217
Static physical load (0–2), mean (sd)	1.41 (0.6)	1.31 (0.5)	1.50 (0.6)	0.463
Psychosocial characteristics				
Social support, (SOS) [1–5], mean (sd)	4.69 (0.5)	4.20 (0.6)	3.81 (0.59)	< 0.001
Catastrophizing, (PCS) [0–52], mean (sd)	9.81 (8.4)	9.07 (8.4)	10.61 (8.4)	0.229
Fear avoidance, (FABQ)				
Physical Activity subscale [0–24], mean (sd)	8.81 (5.5)	7.86 (5.4)	9.99 (5.5)	0.011
Work subscale [0–42], mean (sd)	15.43 (7.9)	14.64 (6.8)	15.89 (8.7)	0.428
Work variables for working population (*n* = 149)				
Full-time work, *n* (%)	111 (74.5)	66.0	72.5	0.412
Working less than 3 y in current job, *n* (%)	41 (27.5)	43.4	24.2	0.016
Work-related complaints (yes), *n* (%)	127 (85.2)	88.3	87.4	0.859
Sickness absence related to CANS				0.185
No, *n* (%)	135 (93.1)	98.1	90.2	
Yes, < 1 mo, *n* (%)	8 (5.5)	1.9	7.6	
Yes, ≥ 1 mo, *n* (%)	2 (1.4)	0	2.2	
Physical workload, (short version PWQ)				
Heavy physical load [7–14], mean (sd)	7.20 (0.7)	7.13 (0.4)	7.26 (0.84)	0.346
Static repetitive load [8–16], mean (sd)	13.65 (2.1)	13.72 (2.4)	13.68 (1.8)	0.929
Psychosocial factors for working population, (JCQ)				
Psychological job demands [5–20], mean (sd)	11.3 (3.0)	11.7 (3.1)	11.0 (2.9)	0.231
Decision authority ([3–12]), mean (sd)	9.5 (1.8)	9.3 (1.6)	9.3 (1.8)	0.246
Skill discretion [6–24], mean (sd)	18.9 (3.0)	18.9 (3.1)	18.9 (2.9)	0.996
Co-worker support [4–16], mean (sd)	11.5 (3.3)	11.9 (3.6)	11.2 (3.1)	0.268
Supervisor support [4–16], mean (sd)	13.2 (2.1)	13.6 (2.2)	13.0 (2.09)	0.176

[..] score range, *sd* standard deviation, *mo* months, *NNGB* Nederlandse Norm Gezond Bewegen (Dutch Norm for Healthy Physical Activity), *VDU* Visual Display Unit, *NRS* Numeric Rating Scale, *DASH* Disabilities of Arm, Shoulder and Hand Questionnaire, *IPA* Impact on Participation and Autonomy questionnaire, *SOS* Social Support Questionnaire, *PCS* Pain Catastrophizing Scale, *FABQ* Fear Avoidance Beliefs Questionnaire, *JCQ* Job Content Questionnaire

^a^Total of groups ‘duration < 3 mo’ and ‘duration ≥ 3 mo’ does not equal total population, due to missing values

^b^means and standard deviations (sd) are presented for continuous scales

Scores on the outcome *perceived participation and autonomy* were low. Restrictions in participation were mainly perceived in the subscales ‘Autonomy outdoors’ and ‘Work and education’. Additionally, Table 1 shows the characteristics within the two subgroups of complaint duration at baseline. Regarding the scores on the outcome measures complaint severity, functional limitations and impact on participation and autonomy, participants with a duration of complaints lasting ≥3 months revealed a higher complaint severity in the previous week, and experienced more functional limitations. Additionally, they perceived a higher impact of their complaints on participation and autonomy, in particular on the subscales ‘Family role’, ‘Autonomy outdoors’, and ‘Work and education’. Although no statistically significant differences were found between the two subgroups, workers in this latter subgroup reported more frequent sick leave due to CANS.

Loss to follow-up is depicted in Fig. 3. Of the 239 participants that provided informed consent, 222 (93%) completed at least the baseline or one follow-up measurement.

### Course of CANS

**Fig. 4:**
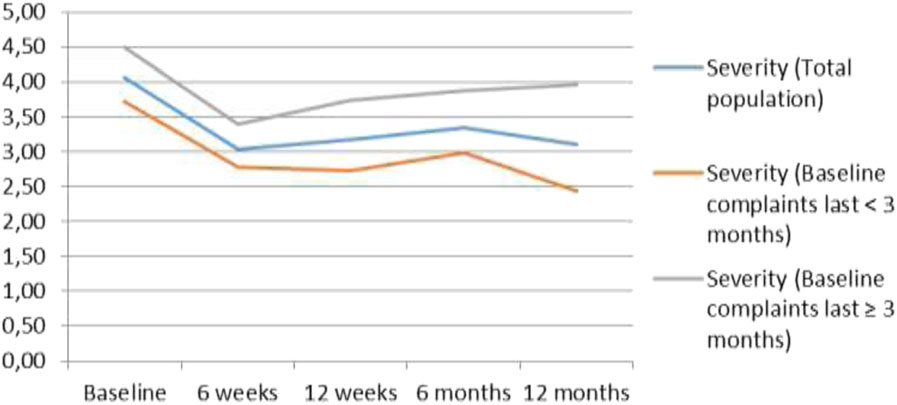
Severity of pain or complaints (NRS) over one-year follow-up

Figure 4 depicts the course of the complaints for the total sample, and for the subgroup ‘duration of complaints < 3 months’ and the course of the subgroup ‘duration of complaints of ≥ 3 months’, for the outcome *severity of complaints in the previous week (NRS-11).* At baseline, the mean score for the total population was 4.1 (SD = 2.3). For the subgroup ‘duration of complaints < 3 months’ the baseline mean score was 3.7 (SD = 2.3), and for the subgroup ‘duration of complaints ≥ 3 months’ the baseline mean score was 4.5 (SD = 2.2). After 1 year, the mean score for the total population was reduced to 3.1 (SD = 2.6). For the subgroup ‘duration of complaints < 3 months’ and the subgroup ‘duration of complaints ≥3 months’, the mean scores were reduced to 2.4 (SD = 2.3) and 4.0 (SD = 2.7) respectively. The group with a longer duration of complaints at baseline showed a relatively smaller reduction in complaint severity, compared to the group with a shorter duration of complaints at baseline. These differences were not significant however.

**Fig. 5:**
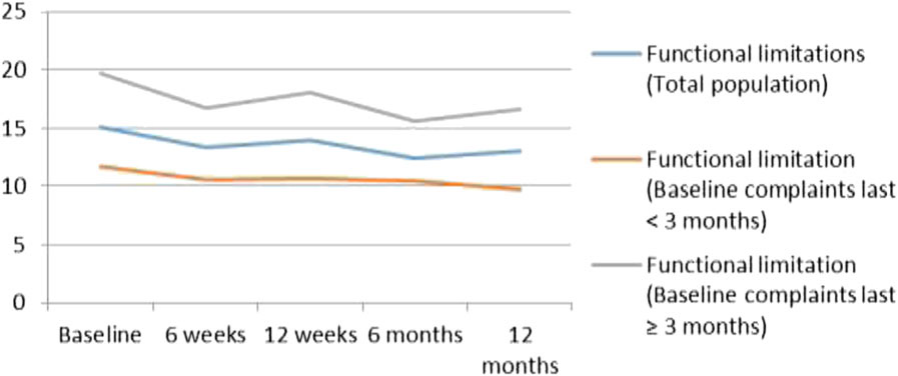
Functional limitations (DASH) over one-year follow-up

Data on *Activities* (DASH) are shown in Fig. 5. The mean score on the DASH for the total population was 15.1 (SD = 12.5) and after 1 year 13.1 (SD = 13.8). For the subgroups ‘duration of complaints < 3 months’ and ‘duration of complaints ≥ 3 months’, the baseline mean scores were 11.6 (SD = 10.6) and 19.7 (SD = 13.6), respectively. After 1 year, the mean DASH scores reduced to 9.7 (SD = 10.7) for the subgroup ‘duration of complaints < 3 months’ and 16.6 (SD = 15.7) for the subgroup ‘duration of complaints ≥3 months’. Both subgroups displayed a small reduction in functional limitations but these differences were not significant.

Differences between baseline scores and scores after one-year follow-up for *Impact on participation and autonomy* are described in Table 2. The baseline scores on all five subscales of the IPA did not change significantly for the total population, nor for the two subgroups on duration of complaints at follow-up. Scores on the IPA at baseline and at 1 year were low, representing minor impact of the complaints on participation and autonomy.

**Table 2:** Scores on participation and autonomy (IPA) for baseline and 1 year follow-up

	Baseline scores, mean (sd)	1 year scores, mean (sd)	*p-value*
Total population			
Autonomy indoors [0–4] (sd)	0.18 (0.33)	0.20 (0.35)	.626
Family role [0–4] (sd)	0.51 (0.59)	0.49 (0.58)	.822
Autonomy outdoors [0–4] (sd)	0.58 (0.62)	0.47 (0.49)	.095
Social life and relationships [0–4] (sd)	0.45 (0.48)	0.45 (0.51)	.968
Work and education [0–4] (sd)	0.86 (0.71)	0.77 (0.65)	.265
Subgroup duration of complaints < 3 mo			
Autonomy indoors [0–4] (sd)	0.15 (0.30)	0.13 (0.26)	.748
Family role [0–4] (sd)	0.38 (0.46)	0.35 (0.43)	.701
Autonomy outdoors [0–4] (sd)	0.47 (0.54)	0.38 (0.41)	.211
Social life and relationships [0–4] (sd)	0.41 (0.45)	0.38 (0.51)	.713
Work and education [0–4] (sd)	0.71 (0.69)	0.63 (0.64)	.473
Subgroup duration of complaints ≥3 mo			
Autonomy indoors [0–4] (sd)	0.23 (0.37)	0.28 (0.42)	.465
Family role [0–4] (sd)	0.66 (0.66)	0.66 (0.69)	.990
Autonomy outdoors [0–4] (sd)	0.69 (0.69)	0.56 (0.56)	.227
Social life and relationships [0–4] (sd)	0.50 (0.52)	0.51 (0.51)	.852
Work and education [0–4] (sd)	1.03 (0.69)	0.92 (0.63)	.355

*[..]* score range, *sd* standard deviation, *mo* months

The results obtained reveal an overall steady pattern of the course over one-year follow-up for all three outcome measures. Generally, the complaints were relatively mild at baseline and did not increase over the one-year follow-up. Because of the overall small change in scores between baseline and measurements at/during one-year follow-up, identification of factors associated with recovery was not feasible. Consequently, these factors could not be investigated. However, the stable pattern raises the question of whether the flat course is genuinely inherent to the study population or whether it may be caused by selection effects. Therefore, the results obtained demanded a closer examination of possible selection effects. The results of this examination will be described in the following section.

### Selection effects

For baseline measurements (time point T0), relevant characteristics of responders to baseline measurements (*n* = 189) were compared with characteristics of participants who did not respond to the invitation to fill out baseline questionnaires (*n* = 50). These non-response analyses for baseline measurements (T0) and their results have been described in detail elsewhere [30]. No significant differences were observed regarding demographics or complaint-related variables between baseline (T0) responders and non-responders.

Table 3 displays the results of the non-response analyses for the time points T1-T4 during follow-up. For the time points T1-T4 during follow-up, differences in baseline variables between respondents and non-respondents were examined, with respect to variables from the domains ‘Health condition’, ‘Personal factors’ and ‘Participation*’*. Differences between both groups were examined by means of independent samples t-tests (continuous variables) and Chi-square/Fisher’s exact tests (categorical variables). *P*-values of < 0.05 were considered to indicate statistical significance.

**Table 3:** Results of the non-response analyses for follow-up measurements

	T1 Responders (n = 180)^a^	T1 Non-responders (n = 56)^a^	*p-value*	T2 Responders (n = 170)^a^	T2 Non-responders (n = 67)^a^	*p-value*	T3 Responders (n = 162)^a^	T3 Non-responders (n = 77)^a^	*p-value*	T4 Responders (n = 165)^a^	T4 Non-responders (n = 74)^a^	*p-value*
	n(%)	n(%)		n(%)	n(%)		n(%)	n(%)		n(%)	n(%)	
Health state (baseline scores)												
Complaints intensity in previous week (NRS) [0–10] mean (sd)	4.18 (2.3)	3.48 (2.2)	0.119	4.06 (2.3)	4.0 (2.3)	0.905	4.13 (2.2)	3.71 (2.16)	0.307	4.05 (2.27)	4.03 (2.31)	0.954
Disability (DASH) [0–100] mean (sd)	15.3 (12.5)	12.6 (12.5)	0.393	15.4 (12.7)	12.3 (10.8)	0.234	14.9 (12.6)	15.5 (12.3)	0.808	14.6 (12.1)	16.9 (14.1)	0.339
Impact participation and autonomy (IPA),												
Autonomy indoors [0–4] mean (sd)	0.21 (0.36)	0.95 (0.12)	0.065	0.18 (0.32)	0.21 (0.41)	0.657	0.16 (0.31)	0.18 (0.27)	0.879	0.16 (0.30)	0.32 (0.41)	0.065
Family role [0–4] mean (sd)	0.50 (0.57)	0.60 (0.79)	0.545	0.52 (0.55)	0.44 (0.83)	0.714	0.49 (0.54)	0.59 (0.50)	0.522	0.46 (0.53)	0.74 (0.82)	0.095
Autonomy outdoors [0–4] mean (sd)	0.57 (0.62)	0.71 (0.58)	0.413	0.57 (0.59)	0.61 (0.86)	0.844	0.53 (0.59)	0.59 (0.47)	0.752	0.55 (0.60)	0.68 (0.68)	0.373
Social life and relationships [0–4], mean (sd)	0.45 (0.48)	0.43 (0.57)	0.879	0.50 (0.48)	0.28 (0.45)	0.096	0.48 (0.50)	0.55 (0.55)	0.621	0.46 (0.48)	0.36 (0.44)	0.323
Work and education [0–4] mean (sd)	0.87 (0.71)	0.80 (0.67)	0.757	0.89 (0.70)	0.59 (0.73)	0.119	0.86 (0.71)	0.96 (0.59)	0.622	0.82 (0.69)	0.83 (0.69)	0.801
Duration of complaints at baseline			0.246			0.371			0.151			0.697
< 3 mo	105 (58.3)	28 (51.9)		95 (55.9)	38 (59.4)		96 (60.0)	37 (50.0)		94 (57.7)	39 (54.9)	
≥ 3 mo	75 (41.7)	26 (48.1)		75 (44.1)	26 (40.6)		64 (40.0)	37 (50.0)		69 (42.3)	32 (45.1)	
History of CANS			0.208			0.151			0.062			0.264
No	20 (15.3)	2 (6.1)		20 (15.5)	31 (88.6)		16 (13.9)	6 (12.2)		15 (12.8)	7 (14.9)	
Yes, one time before	15 (11.5)	2 (6.1)		15 (11.6)	2 (5.7)		16 (13.9)	1 (2.0)		15 (12.8)	2 (4.3)	
Yes, more times before	96 (73.3)	29 (87.9)		94 (72.9)	31 (88.6)		83 (72.2)	47 (85.7)		87 (74.4)	38 (80.9)	
Frequency of discomfort of complaints			0.729			0.862			0.581			0.792
No discomfort	1 (0.8)	0 (0.0)		1 (0.8)	0 (0.0)		1 (0.9)	0 (0.0)		1 (0.8)	0 (0.0)	
Regularly	94 (70.7)	26 (76.5)		93 (71.5)	27 (73.0)		81 (69.8)	39 (76.5)		84 (71.2)	36 (73.5)	
Almost continuously	38 (28.6)	8 (23.5)		36 (27.7)	10 (27.0)		34 (29.3)	12 (23.5)		33 (28.0)	13 (26.5)	
Personal factors												
Gender (female)	133 (73.5)	44 (78.6)	0.555	127 (74.7)	50 (74.6)	0.990	121 (75.6)	56 (72.7)	0.631	123 (75.5)	54 (73.0)	0.683
Age (years), mean (sd)	36.1 (13.4)	33.5 (12.9)	0.193	36.8 (13.5)	32.2 (12.4)	0.014	35.9 (13.4)	34.4 (13.1)	0.421	36.5 (13.8)	33.3 (11.8)	0.069
Paid work	119 (65.7)	28 (50.0)	0.050	114 (67.1)	33 (49.3)	0.017	102 (63.7)	45 (58.4)	0.430	103 (63.2)	44 (59.5)	0.583
Student status	62 (34.3)	28 (50.0)		56 (32.9)	34 (50.7)		58 (36.3)	32 (41.6)		60 (36.8)	30 (40.5)	
Marital status			0.175			0.102			0.728			0.878
Unmarried/ living alone	58 (34.9)	10 (55.6)		54 (34.2)	14 (53.8)		57 (37.7)	11 (33.3)		56 (36.8)	12 (37.5)	
Married/ living together	99 (59.6)	8 (44.4)		95 (60.1)	12 (46.2)		86 (57.0)	21 (63.3)		88 (57.9)	19 (59.4)	
Widow/ divorced	9 (5.4)	0 (0.0)		9 (5.7)	0 (0.0)		8 ()	1 (3.0)		8 (5.3)	1 (3.1)	
Children in the household			0.574			0.753			0.333			0.005
No	144 (86.7)	16 (88.9)		138 (87.3)	22 (84.6)		133 (88.1)	27 (81.8)		137 (90.1)	23 (71.9)	
Yes, one or more	22 (13.3)	2 (11.1)		20 (12.7)	4 (15.4)		18 (11.9)	6 (18.2)		15 (9.9)	9 (28.1)	
Sports participation (yes)	81 (45.0)	27 (50.0)	0.623	77 (45.6)	31 (47.7)	0.441	78 (49.1)	30 (40.0)	0.195	74 (45.7)	34 (47.2)	0.827
Meet Dutch Norm of Healthy Living (yes)	112 (62.2)	37 (68.5)	0.495	110 (65.1)	39 (60.0)	0.282	102 (64.2)	47 (62.7)	0.826	103 (63.9)	46 (63.9)	0.964
Participation												
Sick leave due to CANS			0.036			0.360			0.044			0.357
Yes, < 1 mo	4 (3.4)	4 (14.3)		5 (4.4)	3 (9.1)		4 (3.9)	4 (8.9)		4 (3.9)	4 (9.1)	
Yes, ≥ 1 mo	1 (0.8)	1 (3.6)		1 (0.9)	1 (3.0)		0 (0.0)	2 (4.4)		1 (1.0)	1 (2.3)	
No	114 (77.6)	23 (82.1)		108 (94.7)	29 (87.9)		98 (96.1)	39 (86.7)		98 (95.1)	39 (88.6)	
Full-time work, *n* (%)	86 (68.8)	18 (64.3)	0.811	83 (68.6)	22 (66.7)	0.833	71 (67.9)	32 (68.1)	0.984	76 (71.0)	28 (60.9)	0.217
Working in current job,			0.373			0.929			0.465			0.813
< 3 y	45 (36.0)	7 (25.0)		41 (34.2)	11 (33.3)		38 (35.8)	14 (29.8)		37 (34.6)	15 (32.6)	
≥ 3 y	80 (64.0)	21 (75.0)		79 (65.8)	22 (66.7)		68 (64.2)	33 (70.2)		70 (65.4)	31 (67.4)	
Work-related complaints (yes)	108 (89.3)	21 (80.8)	0.318	104 (89.7)	25 (80.6)	0.147	93 (89.4)	36 (83.7)	0.337	94 (89.5)	35 (83.3)	0.301

*T* Time point, *T1* 6 weeks follow-up, *T2* 12 weeks follow-up, *T3* 6 months follow-up, *T4* 12 months follow-up, *NRS* Numeric Rating Scale, *DASH* Disabilities of the Arm, Hand and Shoulder Questionnaire, *IPA* Impact on Participation and Autonomy Questionnaire, *sd* standard deviation, *mo* month(s), *y* year(s)

^a^Total number of cases does not equal total study population due to missing values on complaint duration

With respect to the domain ‘Health condition’, no statistically significant differences were found between respondents and non-respondents during one-year follow-up. The results within this domain do not indicate a tertiary selection effect in the sense that study participants with more severe complaints, a longer duration of complaints or more hindrance from their complaints are more prone to non-response during follow-up.

Regarding the domain of ‘Personal factors’, the non-response analysis indicated that individuals who did not respond were slightly younger (time point T3), had paid work less often (time point T1 and T2) and more often had young children in the household (time point T5). Regarding the domain ‘Participation*’,* the results of the non-response analyses revealed that individuals who did not respond to one or more of the follow-up measurements displayed more sickness absence due to CANS (time point T1 and time point T3). These latter findings could imply that those who were absent from work because of CANS at the time of baseline measurements or in the preceding 3 months were under-represented in the cohort at several time points during follow-up. It is reasonable to assume that those individuals who were or had been on sick leave because of CANS experienced more severe complaints. Given these findings, it is possible that the observed course of CANS over one-year follow-up might be somewhat less favourable since tertiary selection processes may have taken place, Therefore, some caution is recommended when interpreting the overall results of this study.

## Discussion

### Main findings and interpretation of results

The aim of this prospective cohort study was to describe the characteristics, course and prognostic factors in a screened university population with self-reported CANS, still participating in work or study. In addition, we investigated the mechanism of selective non-response during follow-up and its impact on the interpretation of the results. Unfortunately, due to the overall steady course, the exploration of prognostic factors was not feasible.

Although the outcome measures indicated mild to moderate CANS in terms of the different ICF domains of functioning, the majority of participants indicated perceiving regular or almost continuous discomfort due to their complaints. A previous study revealed that only half of the cohort had previously sought help for their complaints. Of the non-help seekers, 80% indicated having no intention of seeking help in the future [30]. These relatively mild complaints highlight the fact that our study population differs to a great extent from study populations in other studies on the course and prognosis of CANS. The large majority of these studies took place in healthcare settings, resulting in the inclusion of more severe complaints and participants who do display help-seeking behaviour [13, 31–33].

We used the ICF model as a theoretical frame to select measurement instruments in order to describe the *course* in terms of the different domains of functioning. Our findings display a relatively stable course over one-year follow-up and underline the persistent nature of CANS. Other studies investigating the course of CANS, typically performed in healthcare settings, found higher baseline scores on severity and functional limitations [13, 31] and found a significant improvement in mean severity scores and functional disability scores at 3 and 6 months follow-up in the working sub-population. A study by Karels et al. showed that the mean scores on severity and functional limitation reduced approximately by half at 6 months follow-up [13]. Nevertheless, the severity and functional limitation scores in their study still represented mild to moderate limitations in functioning at 6 months follow-up.

Several possible explanations for the relatively stable course that was found in this study may be considered. First, the results could be truly inherent to our study population. Moreover, participants reported a relatively mild complaint severity at baseline, and more than half of the participants reported a duration of complaints shorter than 3 months. According to a recent literature review on CANS, more functional limitations, higher pain intensity at baseline and a longer duration of complaints at baseline are associated with an unfavourable outcome at follow-up [5]. In line with these findings, it was not expected that the complaints would significantly worsen over time. It is noticeable that more than half of the study participants reported a history of CANS. Individuals who have experienced CANS before might be more aware of the possibilities to influence their complaints, or their role in coping with the symptoms [34]. Possibly, they have already adapted their work techniques or learned to avoid certain activities that cause pain. This might have resulted in a reduction in complaint severity or a different perception of limitations in activities.

Second, a methodological issue such as selection effects should be considered as a possible explanation for the results. The manifestation of possible selection effects and the role of these effects in the interpretation of findings will be further discussed in the following section.

### Methodological considerations

A major strength of this study is that the mechanism of potential selection effects has been investigated as much as possible, gaining insight in the validity of the results of this study. However, primary selection effects cannot be ruled out. It is possible that individuals who experience (severe) CANS, choose not to participate in physically or psychologically demanding work or study in the first place, and therefore remain outside the scope of this study. Additionally, the occurrence of secondary selection effects cannot be excluded. It is possible that employees or students have already adapted their work or study activities over time, and are able to adequately cope with their complaints, resulting in a different perception of symptoms or functional limitations.

Furthermore, it is also possible that individuals with high complaint intensity or functional limitations due to CANS have already left the work place or prematurely dropped out of university because of their complaints. As a result, they could not be included in this study cohort in the first place, resulting in an overestimation of the true course.

Lastly, tertiary selection effects might have taken place. Tertiary selection effects could have occurred due to systematic non-response during follow-up. To examine the presence of selection effects during follow-up, non-response analyses were performed for each time point during follow-up (Table 3). Systematic differences between responders and non-responders were examined for a number of relevant factors which were measured at baseline. When interpreting the results of the non-response analyses during follow-up, it should be taken into account that the dataset over one-year follow-up revealed an *arbitrary* missing pattern, i.e. a small proportion of the participants dropped out after filling in one or more of the questionnaires of the follow-up study (monotone missing pattern), and a proportion missed individual measurements during follow-up but returned at a later moment (non-monotone missing pattern) [35, 36].

The results of the analyses of non-response during follow-up revealed no significant differences between responders and non-responders for most variables. It is encouraging that no significant differences were found regarding complaint-related outcomes at baseline. On the other hand, a few variables did reveal indications for the potential presence of selection effects. One of these variables was sickness absence at the time of baseline measurements or in the preceding 3 months. These findings could imply that the observed stable course is mainly based on participants who have not been absent from work because of CANS (and thus possibly perceived less severe complaints). Although these results are based on relatively few participants, the difference between responders and non-responders is significant. Therefore it should be taken into account that the results obtained could be slightly more favourable than the true results of this university population.

These characteristics suggest that we included a group of ‘CANS survivors’ who seem to be able to adequately cope with their complaints. In that sense, this study provides insight in a unique subgroup of individuals with CANS, who do experience a certain level of discomfort and limitations in functioning, but display hardly any help-seeking behaviour or sickness absence.

In the current study, we used an ICF-based model as a theoretical frame to describe the comprehensive concept of functioning over one-year follow-up. Our findings show that a moderate score on one domain of functioning does not automatically result in a moderate score on the other two domains of functioning. By including the three domains of functioning, an overall view of the course of CANS is obtained.

A few methodological limitations of this study need to be considered. First, our sample size was limited. Although in the end we were not able to examine prognostic factors, the small sample size would have hampered the modelling of a wide variety of possible prognostic factors in the first place. Secondly, the optimal study population for follow-up studies is an inception cohort with all individuals in an early stage after onset of the disease. Although we did take the duration of current complaints into account when investigating characteristics and course, it needs to be noted that our study population displayed a variety in complaint duration and the first measurements were therefore not always true baseline measurements.

The current study was aimed as a first step in gaining insight into the course of CANS in a university population and to explore possible prognostic factors. Because of the explorative character of our study and limitations in feasibility, we did not perform multivariate analyses. We encourage future studies in this field who further examine the course and prognosis of CANS to perform multivariate analyses in their statistical approach.

Finally, it needs to be noted that information on treatment during the follow-up period was lacking. Information on treatment would have been valuable in order to determine to what extent the follow-up period reflected the natural progress of the complaints. Nevertheless, it is considered unlikely that many study participants underwent treatment since help-seeking behaviour was largely absent at baseline measurements [30]. Consequently, it is reasonable to assume that the follow-up period primarily reflects the natural course of CANS and is not altered by treatment effects.

### Implications for research and clinical practice

It is often assumed that CANS require a timely preventive intervention in order to prevent beginning and mild complaints from becoming severe and disabling. However, our findings at group level reveal no severe complaints at baseline and an overall stable pattern in terms of all three ICF domains over one-year follow-up. Although the interpretation of these findings requires some caution for the reasons discussed above, they might have important implications for future preventive strategies for CANS in a screened university population. An early preventive intervention for CANS is only worthwhile if there is scope for potential health benefits compared to no early preventive intervention. However, the longitudinal results of our study might question the value of an early preventive intervention in this population, since complaints are mild at baseline and do not seem to worsen at group level. Furthermore, based on the results of a previous study [30], it appears that a considerable number of individuals with CANS appear to be able to make a sound self-assessment of the severity of their complaints and the necessity to seek help. An early preventive intervention appears only to be of added value for those who experience severe hindrance and perceive more severe disabilities due to CANS, but who display no help-seeking behaviour despite the severity of their complaints. Our previous study revealed that this is only a relatively small group from the total group of screened individuals who experience CANS [30]. Consequently, a critical consideration of the necessity of an early preventive intervention (such as an *indicated* preventive intervention) in a university population is encouraged, since the majority of individuals with CANS do not seem to be at risk for the development of severe and disabling complaints over time.

This study was performed in a general university population, which resulted in a relatively young study population (the mean age was approximately 35 years (SD = 13.3 years)). In populations with a higher age, the course of the complaints may be less favourable. Further research is needed to examine the generalisability of our study results in other populations, for example the general population.

Furthermore, future studies should investigate whether the results of this study are also valid in certain subgroups, or whether they can be generalised to other occupational populations. For example, in work settings or fields of study with higher physical work demands (i.e. music academy students/musicians) CANS might be perceived as a serious threat for the ability to perform. In these populations, the symptoms and influence of the complaints on functioning could be rated as more severe [37], and the course of the complaints could be different. Possibly, adequate screening and an early preventive intervention for CANS would result in significant health benefits within certain high-risk groups. When investigating course and characteristics in other screened populations, it is recommended to investigate the presence of selection effects more extensively. For example, by obtaining information during the follow-up period on sickness absence or workplace adjustments due to CANS as much as possible, in order to gain more insight in the manifestation of selection effects.

## Conclusion

In conclusion, this study reveals an overall steady course in this screened population regarding the outcomes linked to the three ICF domains ‘Body functions and structures’, ‘Functioning’ and ‘Participation’ over one-year follow-up. The relatively mild CANS at baseline measurements do not appear to deteriorate over one-year follow-up. However, the results must be interpreted with some caution, since the manifestation of selection effects cannot be ruled out. Since the analyses of non-response during follow-up revealed indications for the potential presence of tertiary selection effects, the true course of CANS might be somewhat less favourable than the results initially suggest. Nevertheless, based on the overall results of this study, it is reasonable to assume that CANS do not deteriorate significantly over one-year follow-up in a screened university population. Therefore, the results of this study could question the necessity of an early preventive strategy in a screened population, largely still participating in work or study.

## Abbreviations

BMI: Body Mass Index
CANS: Complaints of Arm, Neck and/or Shoulder
DASH: Disabilities of Arm, Shoulder and Hand questionnaire
DMQ: Dutch Musculoskeletal Questionnaire
FABQ: Fear Avoidance Beliefs Questionnaire
ICF: International Classification of Functioning, Disability and Health
IPA: Impact on Participation and Autonomy questionnaire
JCQ: Job Content Questionnaire
NRS: Numeric Rating Scale
PCS: Pain Catastrophizing Scale
SOS: Social Support Scale
VDU: Visual Display Unit
